# Severe Pulmonary Cryptococcosis in an Immunocompetent Patient Presenting With Acute Respiratory Failure

**DOI:** 10.7759/cureus.111726

**Published:** 2026-06-29

**Authors:** Briccio Cadiz, Ananya Ganesan, Aditi Iyer, Riley Bricker, Chandra Dommaraju, Praveen Datar, Vikram Oke, Karthik V Iyer

**Affiliations:** 1 Internal Medicine, Mercy Hospital Jefferson, Festus, USA; 2 Internal Medicine, Lake Erie College of Osteopathic Medicine, Erie, USA; 3 Internal Medicine/Infectious Disease, Mercy Hospital Jefferson, Festus, USA; 4 Internal Medicine/Pulmonary and Critical Care Medicine, Mercy Hospital Jefferson, Festus, USA; 5 Internal Medicine/Critical Care Medicine, Mercy Hospital Jefferson, Festus, USA

**Keywords:** acute hypoxemic respiratory failure, atypical pneumonia, bronchoscopy, fungal pneumonia, pulmonary cryptococcus

## Abstract

Pulmonary cryptococcosis may present with nonspecific respiratory symptoms and, in patients with underlying reactive airway disease, can act as a trigger for refractory asthma exacerbation leading to acute respiratory failure, even in immunocompetent individuals. A 60-year-old immunocompetent male, with a negative HIV test, no chronic immunosuppressive therapy, and a remote brief corticosteroid course well below the threshold for clinically significant immunosuppression, presented with approximately two months of progressive dyspnea and cough after multiple treatment courses for presumed asthma exacerbations and bronchitis without improvement. His condition deteriorated during hospitalization, requiring noninvasive ventilation followed by endotracheal intubation for acute hypoxic respiratory failure driven by a refractory asthma exacerbation. The clinical presentation closely mimicked bacterial pneumonia superimposed on asthma exacerbation, with CT imaging demonstrating bronchial wall thickening and mucous plugging, which initially precluded suspicion of a fungal etiology. Serum cryptococcal antigen was negative. An extensive noninvasive infectious workup remained unrevealing, and broad-spectrum antibiotics and bronchodilator therapy failed to produce improvement. On hospital day five, bronchoscopy with bronchoalveolar lavage (BAL) was performed, given persistent hypoxemia and diagnostic uncertainty, with BAL fungal culture growing *Cryptococcus neoformans* and establishing pulmonary cryptococcosis as the underlying trigger of the refractory asthma exacerbation. CNS dissemination was excluded clinically on the basis of intact mentation and absent neurological symptoms throughout the hospital course. Infectious disease consultation determined that respiratory failure was attributable to cryptococcal-triggered asthma exacerbation rather than disseminated disease, and fluconazole monotherapy was initiated accordingly. The patient was successfully extubated on hospital day six and demonstrated steady clinical improvement over the following week with high-flow oxygen support via vapotherm before being discharged on long-term fluconazole. This case highlights the importance of considering fungal etiologies as triggers of refractory asthma exacerbation and emphasizes early bronchoscopy with BAL when noninvasive evaluation is unrevealing in patients with persistent unexplained airway disease.

## Introduction

Pulmonary cryptococcosis is an invasive fungal infection caused by Cryptococcus species, most commonly *Cryptococcus neoformans* and *Cryptococcus gattii*. While classically associated with immunocompromised states, including HIV/AIDS, solid organ transplantation, hematologic malignancy, and prolonged corticosteroid use, increasing evidence demonstrates its occurrence in immunocompetent individuals, often as isolated pulmonary disease [1-4]. In large case series, immunocompetent patients have accounted for a substantial and growing minority of pulmonary cryptococcosis diagnoses; however, severe presentations with acute respiratory failure remain uncommon in this population and carry significant diagnostic and therapeutic challenges [2,3].

Risk factors for pulmonary cryptococcosis in otherwise immunocompetent hosts are not fully established, but proposed contributors include environmental exposure to pigeon droppings or decaying wood harboring *C. neoformans*, subtle immune dysregulation not captured by standard testing, and underlying structural or reactive airway disease [4]. The presence of underlying reactive airway disease may be a particularly relevant risk factor, as fungal airway inflammation can precipitate severe bronchospasm and asthma exacerbation, driving respiratory deterioration out of proportion to what would be expected from the infectious burden alone. In immunocompetent patients, presentations are frequently nonspecific, mimicking bacterial pneumonia, organizing pneumonia, malignancy, or eosinophilic lung disease, and when superimposed on reactive airway disease, may present primarily as a refractory asthma exacerbation, further obscuring the underlying fungal etiology [1-4].

Diagnosis relies on imaging, microbiologic testing, and often invasive procedures such as bronchoscopy with bronchoalveolar lavage (BAL). Specific diagnostic tools include fungal culture, cytology demonstrating encapsulated yeast forms, serum and CSF cryptococcal antigen (CrAg) testing, and emerging metagenomic next-generation sequencing (mNGS) platforms applied to BAL fluid, which have demonstrated promising diagnostic utility in cases where conventional methods are unrevealing [1-5]. Importantly, serum CrAg may be negative in isolated pulmonary disease, and a negative result should not be used to exclude cryptococcosis in patients with high clinical suspicion [6,7]. Management varies based on disease severity and the presence or absence of dissemination, with fluconazole recommended for mild to moderate isolated pulmonary disease in immunocompetent hosts and amphotericin B-based induction therapy required for severe disseminated or CNS infection [6-8]. Radiographic findings vary widely, ranging from solitary pulmonary nodules and masses to cavitary lesions, consolidations, and diffuse infiltrates. In immunocompetent hosts, nodular patterns predominate, although diffuse infiltrates and bronchial wall thickening mimicking bacterial pneumonia or acute respiratory distress syndrome may occur in severe disease [1,2,9,10]. We present a case of pulmonary cryptococcosis in an immunocompetent patient with reactive airway disease, in which the fungal infection acted as the trigger for a refractory asthma exacerbation culminating in acute respiratory failure requiring mechanical ventilation, highlighting the diagnostic challenges of this presentation and important considerations in antifungal management.

## Case presentation

A 60-year-old male with a history of hypertension, hypertriglyceridemia, dyslipidemia, coronary artery disease, and reactive airway disease presented with approximately two months of progressive shortness of breath and cough. During this period, he had multiple urgent care and emergency department visits for recurrent asthma exacerbations and was treated with several courses of broad-spectrum antibiotics without improvement. He had received a brief five-day course of prednisone 40 mg in December, approximately three months prior to presentation, which did not meet criteria for clinically significant immunosuppression. He had no known chronic immunosuppressive conditions, no ongoing corticosteroid use, and HIV testing obtained during this admission was negative. His symptoms included a minimally productive cough and worsening exertional dyspnea.

On presentation, he reported worsening respiratory symptoms and subjective fever. Vital signs were within normal limits; however, he appeared tachypneic with accessory muscle use and was noted to be wheezing on examination, consistent with an acute asthma exacerbation. Lung examination revealed decreased breath sounds bilaterally without focal crackles. Given the clinical picture of wheezing and respiratory distress consistent with asthma exacerbation, combined with CT imaging demonstrating findings consistent with superimposed bacterial pneumonia, broad-spectrum antibiotics were initiated alongside bronchodilator therapy. A fungal etiology was not initially suspected given the absence of immunosuppression and the clinical presentation consistent with a typical asthma exacerbation with bacterial superinfection.

His respiratory status continued to deteriorate despite initial management, reflecting the severity of the underlying asthma exacerbation. He initially required noninvasive ventilation and subsequently required endotracheal intubation for acute hypoxic respiratory failure on hospital day one. Arterial blood gas on fraction of inspired oxygen (FiO₂) 55% showed a pH of 7.32, a partial pressure of carbon dioxide (pCO₂) of 43 mmHg, a partial pressure of oxygen (pO₂) of 112 mmHg, a bicarbonate of 22 mEq/L, and an arterial partial pressure of oxygen (PaO₂)/FiO₂ ratio of 204.

Laboratory evaluation revealed leukocytosis (WBC, 11.7 K/µL) with peripheral eosinophilia (8%), which in retrospect may have represented an early clue toward a non-bacterial trigger of the asthma exacerbation (Table 1). Blood cultures, respiratory viral PCR, and sputum cultures were all negative (Table 2). Serum cryptococcal antigen was negative. Chest radiography demonstrated bibasilar atelectasis with a small left pleural effusion (Figure 1). Computed tomography of the chest showed multifocal pneumonia, bronchial wall thickening, mucous plugging, and a small left pleural effusion, findings interpreted as consistent with bacterial pneumonia complicating asthma exacerbation (Figure 2). Electrocardiogram on presentation demonstrated sinus rhythm with lateral ST changes, prompting cardiology evaluation. Workup for non-ST elevation acute coronary syndrome was completed, including echocardiogram demonstrating preserved ejection fraction; the troponin elevation was attributed to demand ischemia in the setting of acute respiratory failure, and the patient was started on aspirin and heparin accordingly (Table 1, Figure 3).

**Table 1:** Pertinent laboratory values at presentation. Hb = hemoglobin; Hct = hematocrit; WBC = white blood cell count; AST = aspartate transaminase; ALT = alanine transaminase; GFR = glomerular filtration rate; BUN = blood urea nitrogen; Na⁺ = sodium; K⁺ = potassium; Cl⁻ = chloride; CO₂ = bicarbonate; CK-MB = creatine kinase MB isoenzyme; PT = prothrombin time; INR = international normalized ratio; PTT = partial thromboplastin time; HbA1c = hemoglobin A1c.

Lab Test	Patient Value	Reference Range	Units of Measurement
Cardiac Biomarkers			
Troponin (Baseline)	843	≤10	ng/L
Troponin T (6 hr)	423	<15	ng/L
Metabolic / Electrolytes			
Sodium (Na⁺)	139	135-145	mmol/L
Potassium (K⁺)	4.4	3.5-5.1	mmol/L
Chloride (Cl⁻)	106	98-107	mmol/L
CO₂	21	22-29	mmol/L
Calcium	8.3	8.8-10.2	mg/dL
BUN	13	6-20	mg/dL
Creatinine	1.13	0.51-0.95	mg/dL
Glucose	132	74-99	mg/dL
Total Protein	6.6	6.6-8.7	g/dL
Albumin	4.0	4.0-5.0	g/dL
Total Bilirubin	0.6	≤1.2	mg/dL
Alkaline Phosphatase	100	35-104	U/L
AST	44	<40	U/L
ALT	26	≤33	U/L
GFR	>60	≥60	mL/min/1.73 m²
Hematology			
Hemoglobin	15.8	11.9-15.1	g/dL
Hematocrit	48.2	38.0-47.0	%
WBC	11.7	4.0-11.0	K/µL
Platelets	303	150-400	K/µL
Neutrophils	59	50%-70%	—
Lymphocytes	23	20%-40%	—
Monocytes	9	2%-8%	—
Eosinophils	8	1%-3%	—
Basophils	1	0%-1%	—
Coagulation Studies			
Protime	14.7	11.4-14.4	seconds
INR	1.1	0.9--1.1	—
PTT	27.3	24.8-38	seconds
Metabolic / Endocrine			
HbA1c	5.6	<5.7	%
Lactic Acid	1.5	<2.0	mmol/L

**Table 2:** Microbiology/ID laboratory results during hospital course. BAL = Bronchoalveolar Lavage, AFB = Acid-Fast Bacilli, cfu = Colony-Forming Units, RSV = Respiratory Syncytial Virus, MRSA = Methicillin-Resistant *Staphylococcus aureus*.

Lab Test	Patient Value/Result
Influenza A/B, RSV, and COVID-19 PCR Panel	Not Detected
Blood Culture	No Growth
MRSA PCR	Not Detected
Sputum Culture with Gram Stain	No pathogens isolated
	4+ Polymorphic WBC present
BAL Respiratory Culture with Gram Stain	<10,000 cfu/mL normal upper respiratory flora
BAL Fungus Culture	Growth of *Cryptococcus neoformans *
BAL AFB Culture with stain	No AFB species isolated
BAL Pneumonia Pathogen PCR Panel	No nucleic acids detected
Cryptococcal Antigen Blood	Negative

**Figure 1:**
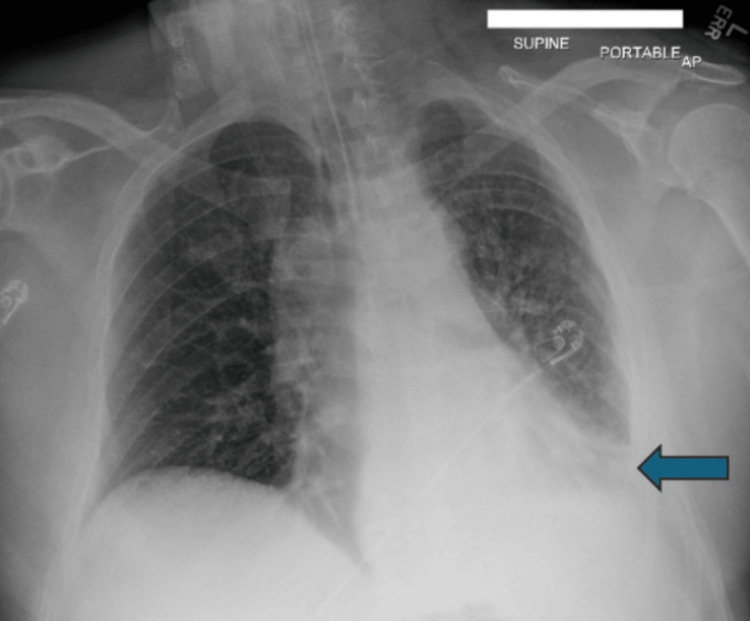
Portable AP chest X-ray demonstrating a small left pleural effusion as indicated by the arrow. AP = Anteroposterior.

**Figure 2:**
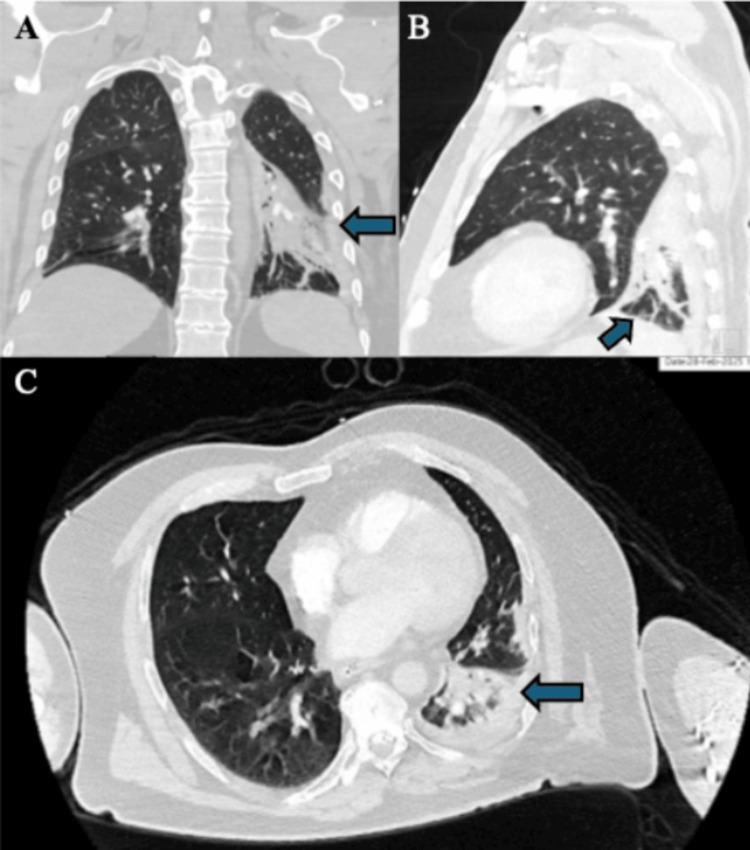
CTA chest with and without contrast taken on day 1 of hospital course. The imaging study revealed left lower lobe pneumonia, bronchitis, mucous plugging, and a small left parapneumonic effusion indicated by the arrows. Select images that best display the significant findings were hand-selected and labeled A-C, with A being a coronal plane image; B being a sagittal plane image; and C being a transverse plane image. CTA = Computed Tomography Angiography.

**Figure 3:**
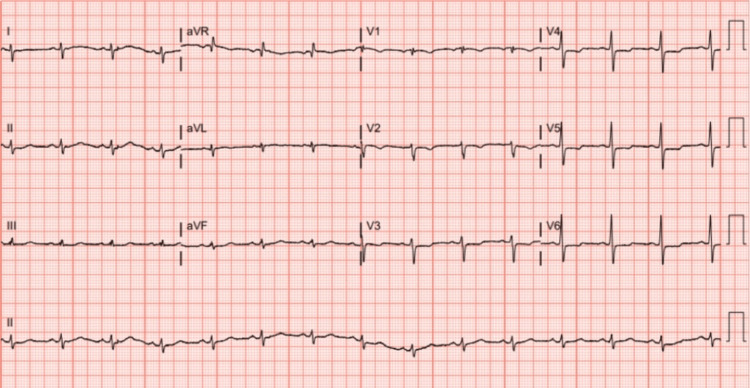
Electrocardiogram (ECG/EKG) at presentation: normal sinus rhythm with lateral ST depressions.

Despite aggressive supportive care including broad-spectrum antibiotics, bronchodilator therapy, and mechanical ventilation, the patient's asthma exacerbation remained refractory with persistent hypoxemia over the following days. A repeat arterial blood gas while receiving FiO₂ 50% demonstrated pH of 7.35, pCO₂ of 40 mmHg, pO₂ of 76 mmHg, bicarbonate of 22 mEq/L, and a PaO₂/FiO₂ ratio of 152, indicating worsening oxygenation. Ventilator settings included assist-control ventilation with a tidal volume of 550 mL, respiratory rate of 16 breaths per minute, and positive end-expiratory pressure (PEEP) of 6 cm H₂O. The failure to improve despite appropriate management for asthma exacerbation and bacterial pneumonia raised concern for an alternative or contributing etiology.

Given persistent hypoxemia, elevated oxygen requirements, and lack of diagnostic clarity despite negative noninvasive testing, bronchoscopy with bronchoalveolar lavage (BAL) was performed on hospital day five (Figure 4). BAL fungal culture grew *Cryptococcus neoformans*, establishing pulmonary cryptococcosis as the underlying trigger of the refractory asthma exacerbation. The patient demonstrated intact mentation and no neurological symptoms throughout his hospital course; CNS dissemination was therefore excluded on clinical grounds without lumbar puncture.

**Figure 4:**
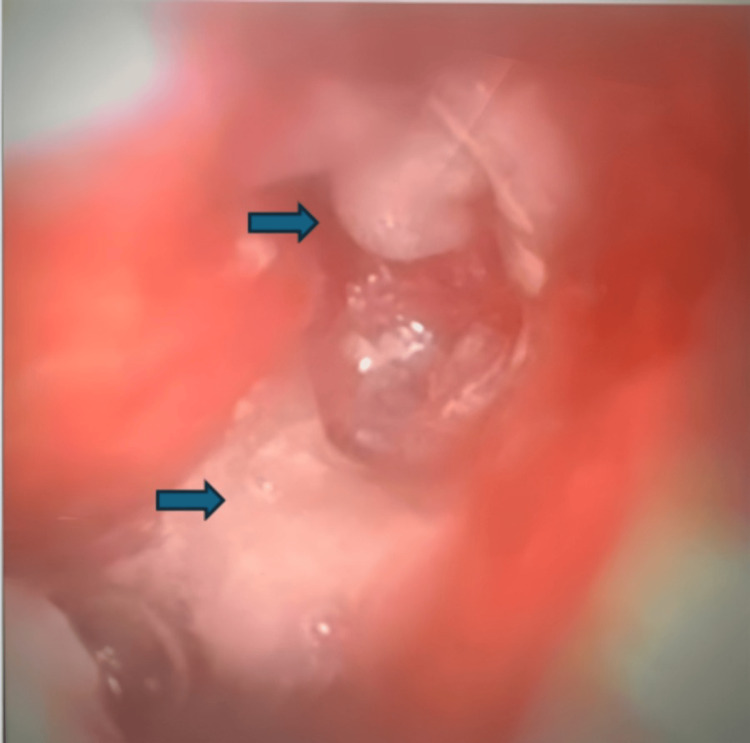
Bronchoscopic image obtained during airway inspection demonstrating inflamed endobronchial mucosa with mucous plugging and whitish plaque-like material at the entrance of the superior segment of left lower lobe bronchial segments. Blue arrows indicate white plaques.

Infectious disease consultation was obtained. The clinical assessment determined that the respiratory failure was driven by a severe asthma exacerbation triggered by pulmonary cryptococcal infection, rather than by invasive or disseminated cryptococcosis. In the setting of an immunocompetent host with isolated pulmonary disease and no evidence of CNS involvement or dissemination, fluconazole monotherapy was initiated on hospital day six in accordance with infectious disease guidance [7]. An initial extubation attempt on hospital day four was unsuccessful, reflecting the ongoing severity of the underlying airway disease. The patient was successfully extubated on hospital day six following initiation of fluconazole. Post-extubation, he continued to exhibit labored breathing and wheezing consistent with residual asthma exacerbation, requiring high-flow oxygen support via vapotherm. However, he demonstrated steady clinical improvement over the following week as the cryptococcal trigger was treated and remained on long-term fluconazole therapy for approximately six months.

## Discussion

Pulmonary cryptococcosis in immunocompetent patients is uncommon but increasingly recognized. In large case series, immunocompetent hosts account for a significant and growing proportion of pulmonary cryptococcosis diagnoses, although severe presentations requiring mechanical ventilation remain rare and are associated with substantial morbidity and mortality [2-4,11]. This case is unusual in that the mechanism of respiratory failure was not direct invasive pulmonary destruction by Cryptococcus, but rather fungal airway inflammation acting as a trigger for severe asthma exacerbation in a patient with underlying reactive airway disease. This distinction has important implications for both diagnosis and management, as the clinical picture closely resembled a refractory asthma exacerbation with bacterial superinfection rather than a primary fungal pneumonia.

The rarity of cryptococcal disease in immunocompetent individuals, combined with the clinical and radiographic overlap with more common conditions, frequently delays diagnosis [3]. An additional and underappreciated contributor to diagnostic delay is the failure to consider Cryptococcus as a potential trigger of refractory asthma exacerbation. Clinicians managing patients with reactive airway disease who fail to improve despite appropriate bronchodilator therapy, corticosteroids, and antibiotics should maintain a broad differential that includes fungal etiologies, particularly in the setting of CT findings atypical for bacterial pneumonia [4]. In this case, the failure of multiple antibiotic courses over two months to produce improvement was the most important clinical signal that an alternative etiology was driving the patient's airway disease.

Risk factors for cryptococcal infection in immunocompetent individuals remain incompletely understood. Proposed contributors include environmental exposures, particularly to pigeon droppings or decaying wood harboring *C. neoformans*, as well as subtle immune dysregulation and underlying airway or structural lung disease [4,11]. In this patient, the presence of reactive airway disease appears to have been the key factor amplifying disease severity, with Cryptococcus acting as an antigenic and inflammatory trigger for bronchospasm and mucous plugging rather than causing diffuse alveolar destruction directly. The CT findings of bronchial wall thickening and mucous plugging, combined with the endobronchial whitish plaques seen on bronchoscopy, are consistent with this mechanism of airway-predominant fungal inflammation.

In retrospect, several findings present earlier in this patient's course may have served as clues toward a fungal trigger of his asthma exacerbation. Peripheral eosinophilia (8%) is a recognized feature of fungal sensitization and allergic bronchopulmonary mycosis, and while nonspecific, its presence alongside refractory airway disease and mucous plugging on CT could prompt earlier consideration of fungal etiologies. Allergic bronchopulmonary aspergillosis is the most commonly recognized fungal trigger of asthma exacerbation, but other fungi including Cryptococcus should be considered in the appropriate clinical context, particularly when standard workup is unrevealing [11]. The endobronchial appearance of inflamed mucosa with whitish plaques on bronchoscopy further supported direct airway involvement by the organism rather than purely parenchymal disease.

Definitive diagnosis in this case required bronchoscopy with BAL, which remains the cornerstone of diagnosis when noninvasive workup is unrevealing [1-3,11]. Notably, serum CrAg was negative in this case, highlighting a critical limitation of this test in isolated pulmonary and airway-predominant disease. While serum CrAg has high sensitivity and specificity for disseminated cryptococcosis and cryptococcal meningitis, its sensitivity is substantially lower in isolated pulmonary disease, and a negative result should not be used to exclude the diagnosis in patients with persistent unexplained airway disease or refractory asthma exacerbation [6,7]. Emerging diagnostic modalities including mNGS of BAL fluid represent a promising avenue for earlier pathogen-agnostic detection in diagnostically challenging cases, with published reports demonstrating successful identification of *C. neoformans* by mNGS in immunocompetent patients where conventional methods were initially unrevealing [12,13]. Earlier bronchoscopy in this case may have shortened the diagnostic delay, reduced ventilator days, and prompted earlier antifungal therapy to address the underlying trigger of the asthma exacerbation.

In cases of severe pulmonary cryptococcosis, evaluation for CNS dissemination is an important consideration given Cryptococcus's well-documented neurotropism [6,7]. In this patient, intact mentation and the complete absence of neurological symptoms throughout the hospital course allowed CNS dissemination to be excluded on clinical grounds. Serum CrAg was also negative, further supporting the absence of disseminated disease. Clinicians should nonetheless maintain a low threshold for lumbar puncture in severe cases, particularly when altered mentation, headache, or other neurological findings are present.

Antifungal management in this case warrants specific discussion. Current Infectious Diseases Society of America (IDSA) guidelines recommend amphotericin B plus flucytosine induction therapy for severe or disseminated pulmonary cryptococcosis [6,7]. However, infectious disease consultation determined that this patient's respiratory failure was driven by a severe asthma exacerbation triggered by pulmonary cryptococcal infection, rather than by invasive or disseminated cryptococcosis itself. In the setting of an immunocompetent host with isolated airway-predominant pulmonary disease and no evidence of CNS involvement or dissemination, the disease did not meet criteria for amphotericin-based induction, and fluconazole monotherapy was initiated accordingly. The patient's favorable clinical response, with successful extubation, resolution of wheezing, and progressive improvement over the following week following initiation of fluconazole, supports this individualized management approach and underscores that treating the underlying fungal trigger was key to resolving the refractory asthma exacerbation. Clinicians managing similar cases should obtain infectious disease consultation early to guide antifungal selection based on host immune status, disease extent, and whether respiratory failure is attributable to direct invasive disease or to fungal triggering of underlying airway disease. For immunocompetent patients with confirmed isolated pulmonary cryptococcosis without dissemination, fluconazole 400 mg daily for six to 12 months is the recommended regimen, with follow-up imaging to confirm radiographic resolution [6,7]. Monitoring for adverse effects including hepatotoxicity and QTc prolongation is warranted during prolonged therapy.

This case underscores two key clinical lessons. First, Cryptococcus should be considered as a potential fungal trigger of refractory asthma exacerbation in immunocompetent patients who fail to improve despite appropriate bronchodilator, antibiotic, and supportive therapy, particularly when peripheral eosinophilia, mucous plugging, or CT findings atypical for bacterial pneumonia are present. Second, a negative serum CrAg does not exclude pulmonary cryptococcosis or cryptococcal airway disease, and bronchoscopy with BAL should be pursued early in patients with persistent diagnostic uncertainty and refractory airway disease.

## Conclusions

Pulmonary cryptococcosis should be considered as a potential fungal trigger of refractory asthma exacerbation and progressive respiratory failure in immunocompetent patients who fail to improve despite appropriate bronchodilator, antibiotic, and supportive therapy. This case illustrates how *Cryptococcus neoformans* can precipitate severe respiratory failure in an immunocompetent host with reactive airway disease by acting as an inflammatory airway trigger rather than through direct invasive pulmonary destruction, closely mimicking bacterial pneumonia superimposed on asthma and significantly delaying diagnosis. A negative serum cryptococcal antigen does not exclude isolated pulmonary or airway-predominant cryptococcal disease, and bronchoscopy with BAL remains essential when noninvasive evaluation is unrevealing. In immunocompetent patients with isolated pulmonary cryptococcosis without CNS involvement or dissemination, in whom respiratory failure is attributable to fungal triggering of underlying airway disease rather than invasive infection, fluconazole monotherapy guided by infectious disease consultation may be appropriate even in the setting of severe respiratory failure requiring mechanical ventilation. These findings should be interpreted in the context of a single case, but serve as an important diagnostic reminder for clinicians managing refractory asthma exacerbation and respiratory failure of unclear etiology.
